# Binding Properties of General Odorant Binding Proteins from the Oriental Fruit Moth, *Grapholita molesta* (Busck) (Lepidoptera: Tortricidae)

**DOI:** 10.1371/journal.pone.0155096

**Published:** 2016-05-06

**Authors:** Guangwei Li, Xiulin Chen, Boliao Li, Guohui Zhang, Yiping Li, Junxiang Wu

**Affiliations:** 1 Key Laboratory of Plant Protection Resources and Pest Management (Northwest A&F University), Ministry of Education, Yangling, Shaanxi, China; 2 Key Laboratory of Applied Entomology, Northwest A&F University, Yangling, Shaanxi, China; 3 Institute of Entomology, Agricultural College, Yangtze University, Jingzhou, Hubei, China; University of Basilicata, ITALY

## Abstract

**Background:**

The oriental fruit moth *Grapholita molesta* is a host-switching pest species. The adults highly depend on olfactory cues in locating optimal host plants and oviposition sites. Odorant binding proteins (OBPs) are thought to be responsible for recognizing and transporting hydrophobic odorants across the aqueous sensillum lymph to stimulate the odorant receptors (ORs) within the antennal sensilla and activate the olfactory signal transduction pathway. Exploring the physiological function of these OBPs could facilitate understanding insect chemical communications.

**Methodology/Principal Finding:**

Two antennae-specific general OBPs (GOBPs) of *G*. *molesta* were expressed and purified *in vitro*. The binding affinities of *G*. *molesta* GOBP1 and 2 (GmolGOBP1 and 2) for sex pheromone components and host plant volatiles were measured by fluorescence ligand-binding assays. The distribution of GmolGOBP1 and 2 in the antennal sensillum were defined by whole mount fluorescence immunohistochemistry (WM-FIHC) experiments. The binding sites of GmolGOBP2 were predicted using homology modeling, molecular docking and site-directed mutagenesis. Both GmolGOBP1 and 2 are housing in sensilla basiconica and with no differences in male and female antennae. Recombinant GmolGOBP1 (rGmolGOBP1) exhibited broad binding properties towards host plant volatiles and sex pheromone components; rGmolGOBP2 could not effectively bind host plant volatiles but showed specific binding affinity with a minor sex pheromone component dodecanol. We chose GmolGOBP2 and dodecanol for further homology modeling, molecular docking, and site-directed mutagenesis. Binding affinities of mutants demonstrated that Thr9 was the key binding site and confirmed dodecanol bonding to protein involves a hydrogen bond. Combined with the pH effect on binding affinities of rGmolGOBP2, ligand binding and release of GmolGOBP2 were related to a pH-dependent conformational transition.

**Conclusion:**

Two rGmolGOBPs exhibit different binding characteristics for tested ligands. rGmolGOBP1 has dual functions in recognition of host plant volatiles and sex pheromone components, while rGmolGOBP2 is mainly involved in minor sex pheromone component dodecanol perception. This study also provides empirical evidence for the predicted functions of key amino acids in recombinant protein ligand-binding characteristics.

## Introduction

The oriental fruit moth *Grapholita molesta* is a highly damaging oligophagous fruit pest. The stone fruit peach (*Prunus persica* L) is considered to be the primary host, and pome fruit pear (*Pyrus communis* L.) and apple (*Malus domestica* L.) are considered secondary hosts [1,2]. The adults can switch from stone to pome fruit orchards during the growing season and cause substantial losses in various host plants [3]. The peach volatiles are only attractive in early and mid season, whereas pear or apple fruit volatiles are attractive from mid to late season [4]. The oriental fruit moth is predominantly guided by olfactory cues towards optimal host plants and oviposition sites [3,5].

Insects recognize semiochemicals in complex environments by mainly relying on their sensitive olfactory systems. In the early events of olfactory processing, the hydrophobic odorant molecules must pass through the aqueous barrier of the sensillum lymph surrounding the dendrites of olfactory receptor neuron (ORNs) cells [6]. Odorant binding proteins (OBPs) are considered to be the first group of proteins that act as carrier molecules in this process [7]. These proteins can selectively capture and transport hydrophobic odorants, as the odor molecules diffuse through the pores on sensilla, through the inner lymph to specific odorant receptors (ORs) localized in the membrane of ORNs [7]. The ORNs can convert chemical signals into an electrical impulse, followed by signal processing in antennal lobes, mushroom bodies and central nervous areas [8]. Finally, the insects make a series of behavioral responses and achieve the recognition of specific semiochemicals. Much previous research has confirmed that OBPs are indispensable in insects’ olfaction perception. *Antheraea polyphemus* abolished the electrophysiological responses when the sensilla lymph containing OBPs *in vivo* was removed. After injecting the sensilla lymph into antennal sensillum again, electroantennogram (EAG) responses to the sex pheromone component (E, Z)-6,11-hexadecadienyl acetate returned to normal [9]. Compared with wild-type of *Drosophila melanogaster*, lush (OBP76a) mutants were completely insensitive to pheromone component 11-*cis* vaccenyl acetate, demonstrating that lush was an essential component in pheromone signal transduction [10]. In addition, RNA interference and site-directed mutagenesis experiments have confirmed that OBPs are essential for the correct functioning of the olfactory system in insects [11–14].

To date, Lepidopteran OBPs are usually subdivided into three main subfamilies; pheromone binding proteins (PBPs), general OBPs (GOBPs), and antennal binding proteins (ABPX) [15–17]. The PBPs are located in the sensilla trichodea and can recognize sex pheromones [18–21]. Moth GOBPs contain two conserved members and are clustered into two separate clades (GOBP1 and GOBP2) in a phylogenetic tree [22,23], suggesting they may have distinct roles in odorant recognition. Unlike the PBPs, both GOBPs are highly expressed in antennae of both sexes with no differences in expression level [15]. Previously, Lepidopteran GOBP distribution was found to be limited to sensilla basiconica, and the function was mainly involved in the detection of interspecific signals such as host plant volatiles and other environmental chemicals [22–24]. However, many studies have indicated that GOBPs located in sensilla trichodea are highly sensitive to sex pheromone components. For instance, MsexGOBP1, MbraGOBP2 and BmorGOBP1 were expressed in sensilla trichodea of male antennae that respond to sex pheromones [25–27]. Similar results were also obtained in AtraGOBP, OachGOBP, SexiGOBP, and ApolGOBP [28–31].

Although numerous experiments have verified that OBPs are essential in olfaction recognition of insects, there are a lot of debates on how OBPs specifically recognize, transport and release odorant molecule stimuli to the odorant receptors (ORs). The NMR structure of *Bombyx mori* PBP1 (BmorPBP1)–pheromone complex demonstrated that a serine residue, Ser56, specifically bonded to bombykol with a hydrogen bond from the hydroxyl group. BmorPBP1 has a long C-terminal which forms a regular helix α7 occupying the pheromone-binding site in the binding pocket at pH 4.5, whereas the helix α7 changed into an extended conformation on the surface of BmorPBP1 at pH 7.0, and the protein exhibited a strong binding affinity for bombykol [32]. Different from BmorPBP1, bombykol bonding to BmorGOBP2 involves hydrogen bonding to Arg110 rather than to Ser56 as found for BmorPBP1 [17]. Analysis of the crystal structures of the *Drosophila melanogaster* protein lush in complexes with short-chain alcohols showed that the Thr57 is the most critical residue for binding ethanol, and the T57A substitution completely abolished binding affinity [33]. Jin et al [34] reported that the ectodomain of sensory neuron membrane proteins (SNMPs) emerges out of the membrane surface and imposes its own electrostatic influence on *Drosophila* PBP leading to pheromone release at the vicinity of the OR, the *Drosophila* PBP was very likely exhibiting distinct mechanisms of ligand release.

We have annotated 28 OBP genes from the female antennae of *G*. *molesta* using RNA-seq sequencing [35]. The binding abilities of six OBPs have been verified along with four sex pheromone components and various host plant volatiles [36–38]. The gene sequences and expression patterns of the two GmolGOBPs have been identified via homologous cloning and real-time quantitative PCR (qRT-PCR), respectively [39]. However, the intracellular localization, binding specificities and other physiological functions are not clear yet.

In this study, we assessed the ligand-binding activities of rGmolGOBP1and 2 using sex pheromone components and host plant volatiles. We also characterized the distribution of GmolGOBP1 and 2 in different sensillum types via immunohistochemical localization. Based on the results of fluorescence binding assays, we combined the homology modeling, molecular docking, site directed mutagenesis and ligand-binding assays to investigate the binding sites of GmolGOBP2 to dodecanol and proposed a possible ligand-binding mechanism.

## Materials and Methods

### Insect rearing

The females of *G*.*molesta* we used were obtained from our laboratory colony that had been reared for more than 60 generations at the College of Plant Protection, Northwest A&F University, Yangling, Shaanxi, China. The larval individuals were originally collected in peach orchards of Yangling in 2008. The owners of the orchard permitted us to collect the samples. The larvae were reared on an artificial diet in the laboratory and kept at 25±1°C, 70%±5% relative humidity (RH) with a day/night cycle of 15:9 [40]. Pupae were sexed, and males and females were placed in separate glass tubes after emergence. The adult moths were fed with a cotton swab, dipped in 5% honey solution and changed daily. For total RNA isolation, the female antennae were collected from three- to four day-old moths and immediately transferred to 1.5 mL Eppendorf tubes immersed in liquid nitrogen and were stored at -80°C prior to use. For fluorescence immunohistochemistry experiments, the antennae were dissected from 4-day-old male and female moths and immediately used to fixation.

### RNA extraction and cDNA synthesis

Total RNA was isolated using RNAiso Plus extraction reagent (TaKaRa, Shiga, Japan) according to the manufacturer’s protocol, the RNA integrity was evaluated using 1.5% agarose gel electrophoresis, and the concentration was quantified by spectrophotometry (SimpliNano, GE, USA). First-strand cDNA was synthesized with Oligo (dT)_18_ anchor primers, using RevertAid^™^ First Strand cDNA Synthesis Kit (Fermentas, MBI) at 42°C for 60 min, and then reaction was terminated by heating at 70°C for 5 min, in accordance with the recommended protocols. The product was immediately used for PCR amplification or stored at −20°C.

### Construction of expression vector

The coding regions of mature GmolGOBP1 and 2 were amplified using gene-specific primers (Table 1). PCR was conducted via initial denaturation at 94°C for 5 min, followed by 30 cycles of 94°C for 30 s, 56°C for 30 s, 72°C for 45 s, then final extension at 72°C for 10 min. The PCR products were separated on agarose gels and purified with a Biospin Gel Extraction Kit (BioFlux, Hangzhou, China), and then cloned into a pMD19-T cloning vector (TaKaRa Co., Dalian, China) by T/A Pairing. Individual positive clones were verified to contain the desired sequences, and pET28a (+) plasmids (Novagen, Madison, WI) were digested using corresponding restriction endonucleases and ligated together with T4 DNA ligase (TaKaRa, Shiga, Japan). Three randomly selected positive pET28a (+)/GmolGOBPs plasmids were sequenced. To express the proteins, the plasmids containing the correct GmolGOBP1 and 2 sequences were extracted and transformed into *E*. *coli* BL21 (DE3) chemically competent cells (Trangen Biotechologies, Beijing, China). For correctly transformed recombinant plasmids, positive clones of GmolGOBP1 and 2 in pET28a (+) were sequenced and ensured to contain the correct gene encoding of the mature proteins.

**Table 1:** Gene-specific primers used for expression analyses of GmolGOBPs and for site-directed mutagenesis of GmolGOBP2 key amino acid residues.

Primer name	Sequence (5'→3')	Product size
**Heterologous expression of GmolGOBP1 and 2**		
GmolGOBP1		
Sense	CGGGATCCACCCAGGAGGTGCTGAA (BamHΙ)	435 bp
Antisense	CCCAAGCTTGGGTCAAGCATCAGCCTCCA (Hind III)	
GmolGOBP2		
Sense	CGGGATCCGGCCGGATGGTAGATGGTAC (BamHΙ)	444 bp
Antisense	CCCAAGCTTGGGTCAGTATTTCTCCAGTACAG (HindIII)	
**Site-directed mutagenesis of GmolGOBP2**		
T9→A9: ACT→GCT		
T9A-Sense	TGGGAAGGCGTTGGAGCAGTGT	3127 bp
T9A-Antisense	AAATGCGCAGCGACATGACTC	
V111→A111: GTG→GCG		
V111A-Sense	ACTGCAGCCGCGCGGTGAAGGT	3127 bp
V111A-Antisense	CGTCAGAGATATCGTCGTATTGTT	
V114→A114: GTG→GCG		
V114A-Sense	TGAAGGCGGCAGCTTGCTTCAAGGT	3127 bp
V114A-Antisense	CCACGCGGCTGCAGTCGTCAGAGA	

The restriction endonucleases are in parentheses after each primer, and the restriction sites are underlined. Mutated nucleotide sites are highlighted in bold.

### Expression and enrichment of rGmolGOBP1 and 2

Each overnight-grown culture was inoculated into 2 L fresh Luria-Bertani (LB) medium with kanamycin (50 μg/ml), and then grown at 37°C until it reached a value of OD_600_ = 0.6. rGmolGOBP1 and 2 were induced by adding isopropyl β-D-1-thiogalactopyranoside (IPTG) (24 mg/ml) to a final concentration of 0.3 mM for 8 h at 28°C. The bacterial cells were harvested via centrifugation (8,000 g, 10 min), then sonicated in lysis buffer (1 mM phenylmethanesulfonyl fluoride, 250 mM NaCl, and 20 mM Tris-HCl pH 7.4) and centrifugated again (12,000 g, 30 min). Sodium dodecyl sulfate polyacrylamide gel electrophoresis (SDS-PAGE) analysis showed that rGmolGOBP1 and 2 were all presented as inclusion bodies. To obtain the solubilized proteins, the inclusion bodies were solubilized and refolded following the protocol of previous studies [21,41]. The supernatant of the rGmolGOBP1 and 2 was enriched by Ni-NTA His·Bind Resin column (7sea Pharmatech Co., Shanghai, China). The purity and concentration of the soluble proteins were detected using SDS-PAGE and BCA protein assay kit (Beyotime, Shanghai, China), respectively.

### Preparation of Antisera

The GmolGOBP1 and 2 antigens were provided as unconjugated recombinant proteins in Freund’s complete (initial immunization) and Freund’s incomplete (further immunization) adjuvant at a ratio of 1:1. Antisera of each protein were raised by injecting the corresponding antigen into two adult male rabbits subcutaneously and intramuscularly with 400–500 μg enriched GOBP for each rabbit on days 1, 14, 27 and 40. The adult male New Zealand white rabbits for research were purchased from Laboratory Animal Husbandary Company of Yangling, Shaanxi, China. Their weight ranged from 2 to 3 kg. Laboratory rabbits were also reared by the same company after each antigen injection. The rabbits were reared in a standard experiment animal room. Temperature ranged from 20 to 25°C and ventilation frequency was 10 times/d. The noise was less than 60 dB with a day/night cycle of 12:12. The rabbits fed on artificial diet and got food and water freely. The animals were bled on the tenth day after the last injection and the blood was centrifuged at 3000 rpm for 5 min at 4°C. The supernatant antisera were pooled and stored at -20°C. Pre-immune serum was collected from each respective rabbit before the initial immunization. The titer of the antibody against GOBP was determined by ELISA after the second and the last immunizations. After the test, rabbits were anesthetized with soluble pentobarbital sodium (30 mg/kg, mass ratio of anesthetic and rabbit) via vein of ear margin, and then performed euthanasia by injecting 20 ml air into entotic vein. All animal work was performed in conformity with institutional guidelines for the care and use of laboratory animals, and protocols were approved by the Institutional Animal Care and Use Committee in Northwest A&F University, Yangling, Shaanxi, China.

### Immunocytochemical localization

We used WM-FIHC to visualize GmolGOBP1 and 2 with the specific antibodies in the antennal sensilla. In order to improve antibody penetration into tissues as well as immunostaining effectiveness, all incubations and washes were performed in a volume of 0.25 ml in thin walled PCR tubes (Kisker, Germany) applying slow rotation or moderate shaking. Antennae were dissected from cold anesthetized moths and immediately transferred to PCR tubes immersed in ZnFA fixation solution (1% formaldehyde, 0.25% ZnCl_2_, 1.2% sucrose, 135 mM NaCl, 0.03% Triton X-100) for 24 hours at room temperature. The fixation solution was removed, and the antennae were washed thrice for 10 min each with an HBS buffer (10 mM hepes, 25 mM sucrose, 150 mM NaCl, 5 mM KCl, 5 mM CaCl_2_, 0.03% Triton X-100). Then the antennae were carefully squeezed about fifteen times with a fine point forceps in the same HBS buffer under binocular inspection. Another wash followed after causing small cracks in the antennae cuticle. Subsequently, the antennae were incubated for two hours in 20% dimethyl sulfoxide (DMSO) / 80% methanol, washed for 10 min in 0.1 M Tris-HCl pH 7.4, 0.03% Triton X-100. Then the antennae were incubated in a blocking solution (PBS, 1% DMSO, 5% albumin bovine V, 0.03% Triton X-100) for 5 hours at room temperature. The solution was substituted by the same blocking solution containing the primary antibody diluted 1:500, and the tubes were placed for 15 sec in a water bath sonifier and then incubated for 5 days at 4°C, repeating the sonification once a day. The control experiments were performed with pre-immune serum diluted 1:500 instead of the primary antibody. After four washes in PBS, 1% DMSO, 0.03% Triton X-100 for 15 min each, the antennae were treated with an anti-Rabbit goat 488-AffiniPure secondary antibody in blocking solution, and ultrasonically treated for 15 sec in a water bath sonifier and then incubated for 5 days at 4°C in the dark. Finally, the antennae were washed in PBS with 1% DMSO, 0.03% Triton X-100 four times for 15 min each before they were mounted in mowiol solution. Immunolocalization of GmolGOBP1 and 2 were analyzed with an inverted fluorescence microscope (Zeiss, Oberkochen, Germany).

### Fluorescence binding assays

Fluorescence binding assays were performed on a Hitachi (Tokyo, Japan) F-4500 fluorescence spectrophotometer with a 1 cm light path fluorimeter quartz cuvette. Both of the slit widths for excitation and emission were 10 nm. 1-N-phenyl-naphthylamine (1-NPN) was dissolved in HPLC purity grade methanol to yield a 1 mM stock solution. A 2 μM solution of the protein in 20 mM Tris-HCl, pH 7.4, was titrated with aliquots of 1 mM 1-NPN to final concentrations of 1, 2, 4, 6, 8, 10, 12, 14, 16, 18, and 22 μM. The probes were excited at 337 nm and emission spectra were recorded between 370 and 550 nm. All ligands (S1 File) used in competitive binding assays were also dissolved in HPLC purity grade methanol. The final concentrations of sex pheromones were between 1 and 22 μM for each rGmolGOBP protein, whereas the concentrations of 30 synthetic plant volatiles to rGmolGOBP1 and 2 were up to 64 and 100 μM, respectively. Binding data were collected in three independent measurements.

Dissociation constants of the complex GmolGOBPs/1-NPN (K_1-NPN_) were calculated by a nonlinear regression binding curve, using Prism 5.0 (GraphPad Software Inc.). The inhibition constants (K_i_) of the competitors were computed from the corresponding IC_50_ values using the equation: *K*_*i*_ = [IC_50_]/(1+[1−NPN]/K_*1−NPN*_), where [1−NPN] is the free concentration of 1−NPN and K_1-NPN_ is the dissociation constant of the complex protein/1-NPN.

### Effect of pH on binding affinity of rGmolGOBP2

Based on the fluorescence measurement experiments as described above, we further evaluated the effects of pH on the binding affinities of GmolGOBP2 to ligands; the binding of GmolGOBP2 to the representative ligand dodecanol was detected at six different pH values (4.0, 5.0, 6.8, 7.4, 8.0 and 8.8) in 20 mM Tris-HCl buffer. All the values of binding affinities were collected in three independent measurements. Data are reported as means±SEM. Difference in pH effect on binding affinity of rGmolGOBP2 was analyzed with one-way analysis of variance (ANOVA), followed by the Student-Newman-Keuls (S-N-K) test at α = 0.05. P<0.05 was considered statistically significant. For statistical analysis we used SPSS 16.0 software (SPSS Inc., Chicago, IL).

### Three-dimensional (3D) structure modeling and molecular docking

The amino acid sequence (without signal peptide) of GmolGOBP2 was submitted to the Meta Serve (http://www.bioinfo.pl/meta/) to find structural homologues. Based on sequence similarity, we selected the crystal structure of the *Bombyx mori* general odorant binding protein (BmorGOBP2, PDB code: 2wck.1) as the template to build the 3D structure of GmolGOBP2 [17]. Using Modeler module in Discovery Studio 2.0 (Accelrys Software Inc.) [42], various initial 3D models were constructed, and the one with the highest score of Profiles-3D was chosen. The initial homology model was further refined following the published protocol [8,43]. The non-bond list radius of 14.0 Å was used; non-bond higher and lower cutoff distances were 12.0 and 10 Å, respectively. Using the most optimized 3D model, potential binding modes of GmolGOBP2 to dodecanol were defined and further edited by the docking program CDOCKER [44]. The top 20 docking poses ranked by CDOCKER energy were retained for subsequent findings of the most optimal binding mode.

### Site-directed mutagenesis and expression of mutants

Gene-specific primers were designed in accordance with the recommended protocols of the TaKaRa MutanBEST Kit (TaKaRa, Shiga, Japan). The GmolGOBP2 coding sequence was mutated to create the three mutants GmolGOBP2 T9A (threonine to alanine at position 9), GmolGOBP2 V111A (valine to alanine at position 111), and GmolGOBP2 V114A (valine to alanine at position 114). Mutations were obtained using the TaKaRa MutanBEST Kit with *Pyrobest* DNA polymerase with the pMD19-T/GmolGOBP2 plasmid DNA as template. All plasmids containing mutations were verified by DNA sequencing. Mutated protein expression and enrichment were performed as described above for rGmolGOBP1 and 2. After determining concentrations, purified mutants were applied to verify the binding affinities with dodecanol and its analogs in fluorescence ligand-binding assays.

## Results

### Expression and enrichment of rGmolGOBP1 and 2

**Fig 1:**
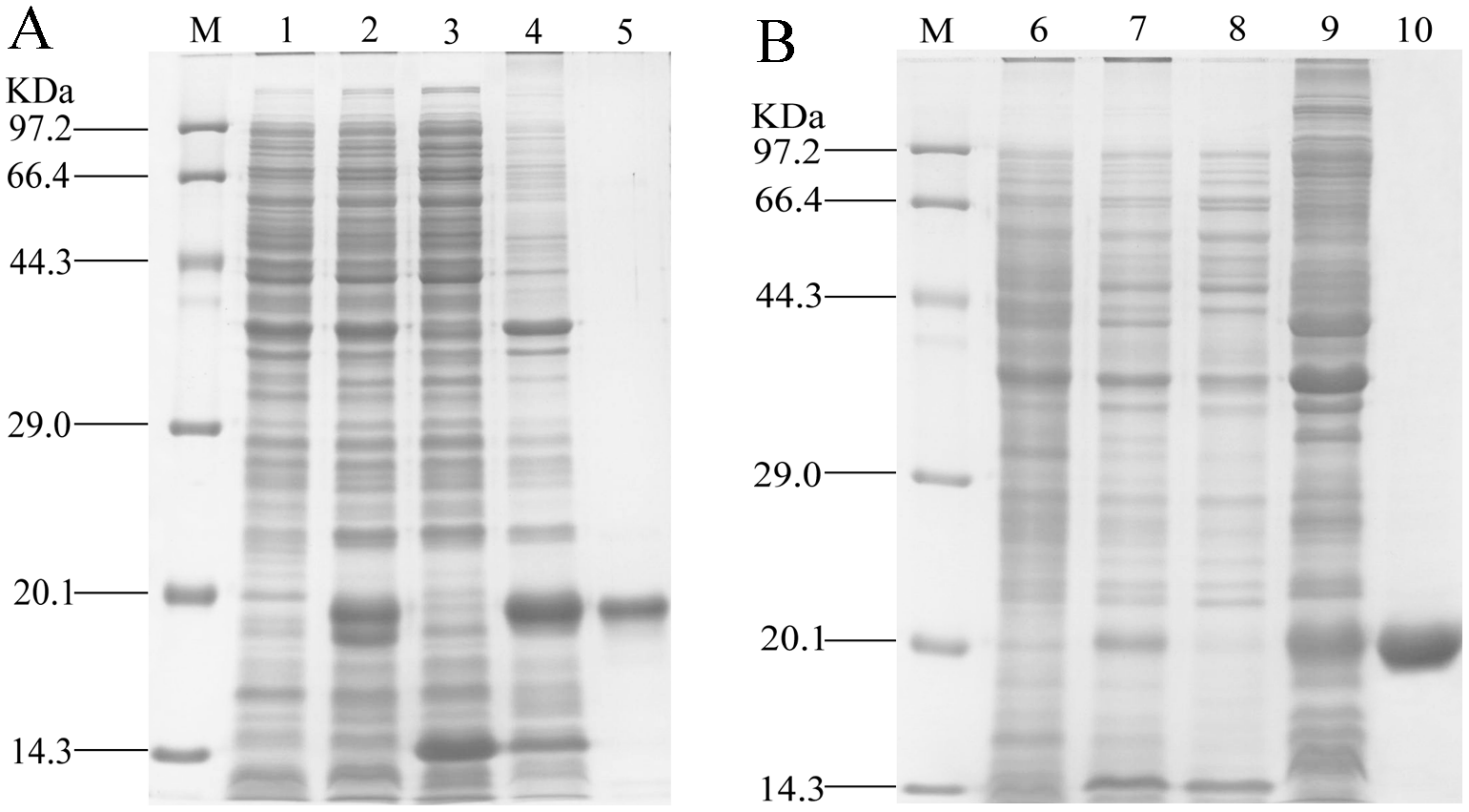
SDS-PAGE analysis of rGmolGOBP1 (A) and rGmolGOBP2 (B) in *Escherichia coli*. M. Protein molecular weight marker; 1. Non-induced pET28a(+)/GmolGOBP1transformed BL21 (DE3) cells; 2. Induced pET28a(+)/GmolGOBP1transformed BL21 (DE3) cells; 3. Supernatant of disrupted pET28a(+)/GmolGOBP1 IPTG induced cells; 4. Inclusion body of disrupted pET28a(+)/GmolGOBP1 IPTG induced cells; 5. Purified rGmolGOBP1; 6. Non-induced pET28a (+)/GmolGOBP2 transformed BL21 (DE3) cells; 7. Induced pET28a(+)/GmolGOBP2 transformed BL21 (DE3) cells; 8. Supernatant of disrupted pET28a(+)/GmolGOBP2 IPTG induced cells; 9. Inclusion body of induced pET28a(+)/GmolGOBP2 IPTG induced cells; 10. Purified rGmolGOBP2.

The rGmolGOBP1 and 2 were expressed successfully in prokaryotic expression system. Analysis of SDS-PAGE showed that two rGmolGOBPs were present as inclusion bodies (Fig 1). The proteins were solubilized by denaturation in Guanidine hydrochloride (GuHCl)/DTT and renatured by extensive dialysis against 20 mM Tris-HCl buffer, pH 7.4. Concentrations of the enriched rGmolGOBP1 and 2 were 1.14 and 0.52 mg/ml, respectively. The molecular weights of the final enriched rGmolGOBP 1 and 2 were all about 20 kDa, which were consistent with the theoretical molecular weight (Mw) of 20.2 and 20.0 kDa calculated by isoelectric point/Mw online program (http://web.expasy.org/compute_pi/) (Fig 1).

### Immunolocalization of GmolGOBP1 and 2

**Fig 2:**
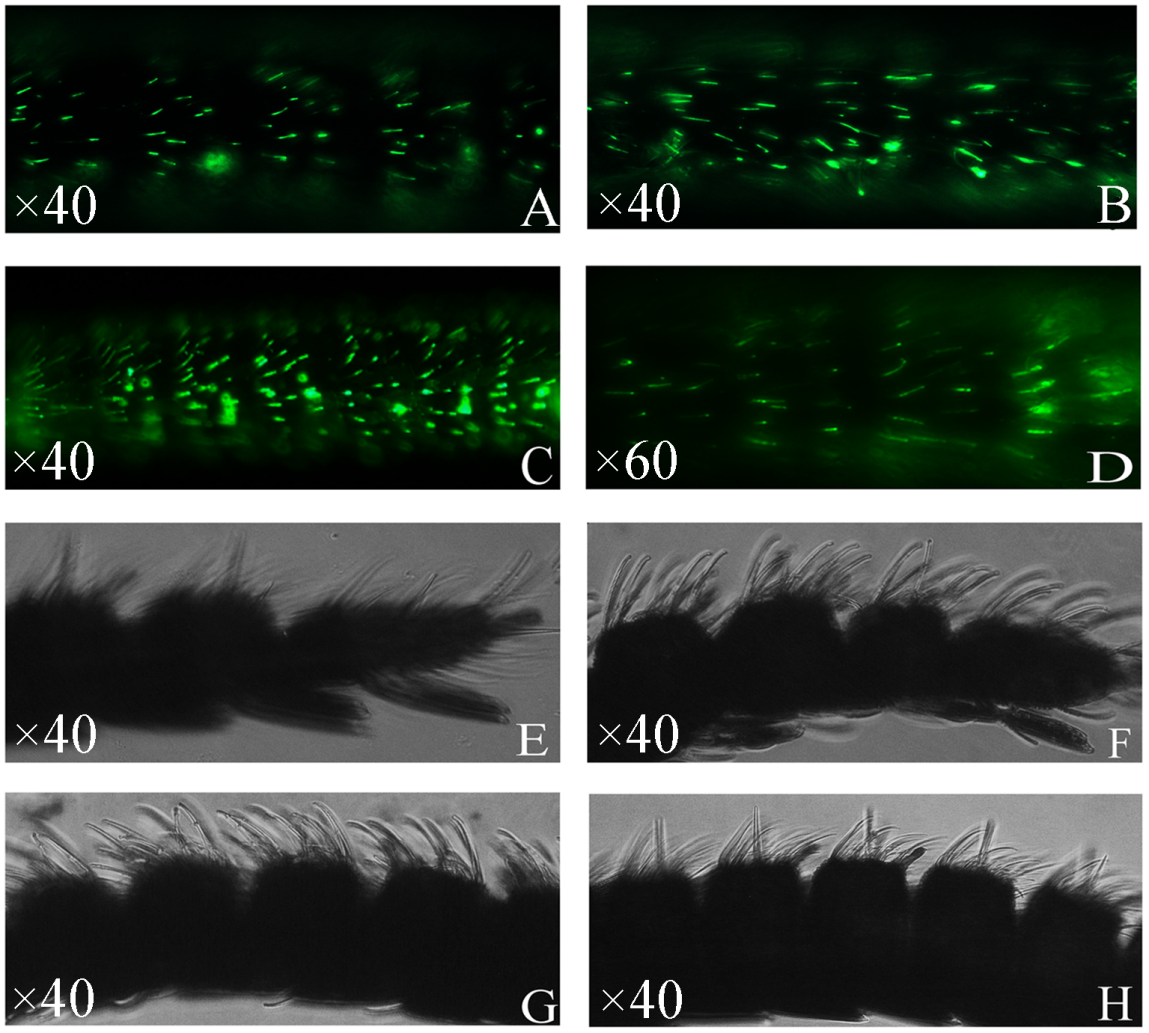
Immunolocalization of GOBP1 and 2 in the antennae of *G*.*molesta*. Whole mount preparations were probed with antisera specific for GmolGOBP1 (A. female, B. male) and GmolGOBP2 (C. female, D. male). Immunoreactivity was visualized by 488-AffiniPure secondary antibody. A high number of GmolGOBP1 and 2-expressing cells are visible and can be located in sensilla basiconica. E (female), F (male) and G (female), H (male) represent control experiments with GmolGOBP1 and 2 pre-immune serums producing no labeling, respectively.

To define the sensillum type housing GmolGOBP1 and 2, we employed antisera generated against GmolGOBP1 and 2 in WM-FIHC experiments. After the last immunization, the titers of the antibody against GmolGOBP1 and 2 were 1:320000 and 1:500000, respectively. The results strongly indicated that a subpopulation of the antennal sensilla basiconica was intensively labeled with GmolGOBP1 and 2 (Fig 2A–2D). Similar control experiments with the respective pre-immune serum observed non-labeling (Fig 2E–2H). The number of labeled s. basiconica was significantly higher for anti-GmolGOBP2 compared to anti-GmolGOBP1 (Fig 2A–2D).

### Fluorescence binding assays

**Fig 3:**
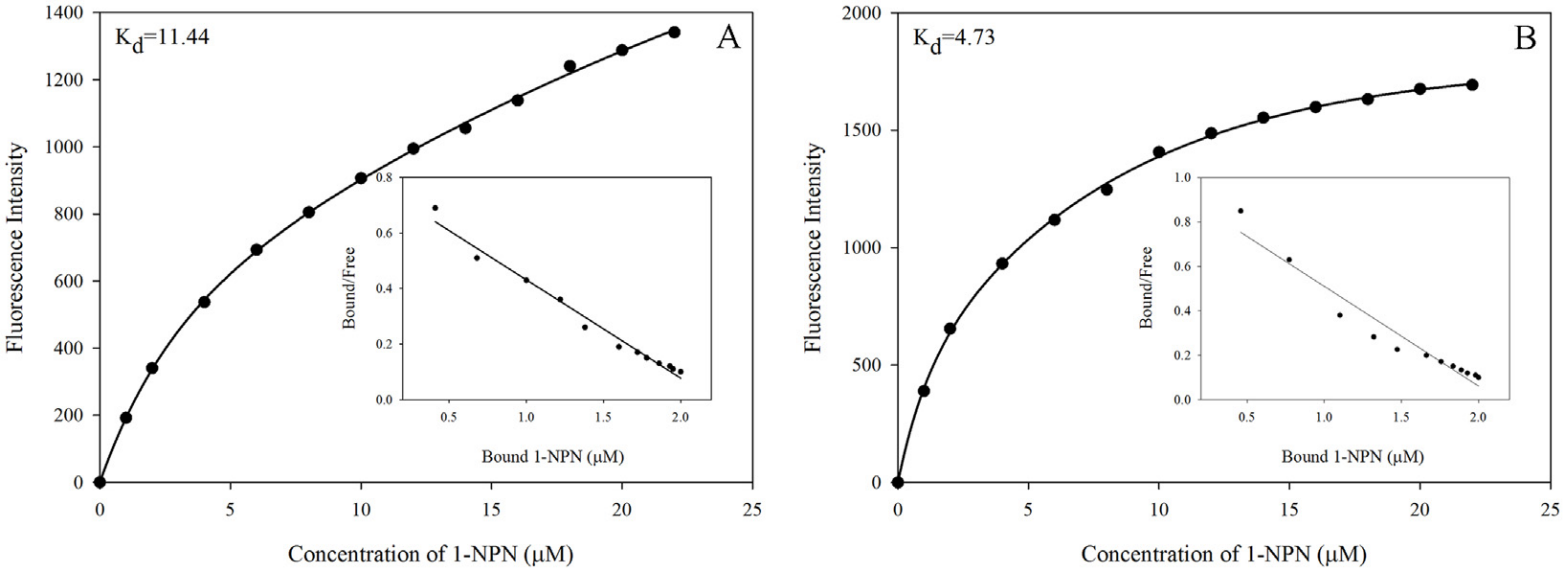
Binding curves of 1-NPN and relative Scatchard plots for rGmolGOBP1 and 2. A. rGmolGOBP1; B. rGmolGOBP2. A 2 μM solution of each protein in 20 mM Tris-HCl buffer (pH 7.4) was titrated with a 1 mM 1-NPN solution in spectrophotometric-grade methanol to final concentrations of 2 μM to 22 μM, and the emission spectra were recorded between 370 and 550 nm. The dissociation constants of rGmolGOBP1 and 2 were 11.44 and 4.73 μM, respectively.

The ligand-binding affinities of two enriched rGmolGOBPs were evaluated using four sex pheromone components and 30 host plant volatiles. When excited at 337 nm, in presence of 1-NPN both GmolGOBPs showed a strong emission spectrum shift from 460 nm to approximately 400–410 nm, as well as a drastic increase in fluorescence intensity, suggesting the 1-NPN was suitable for investigating odorant ligand binding in fluorescent displacement assays. Titration of rGmolGOBP1 and 2 with increasing concentrations of 1-NPN allowed measurement of dissociation constants of 11.44 and 4.73 μM, respectively (Fig 3).

**Fig 4:**
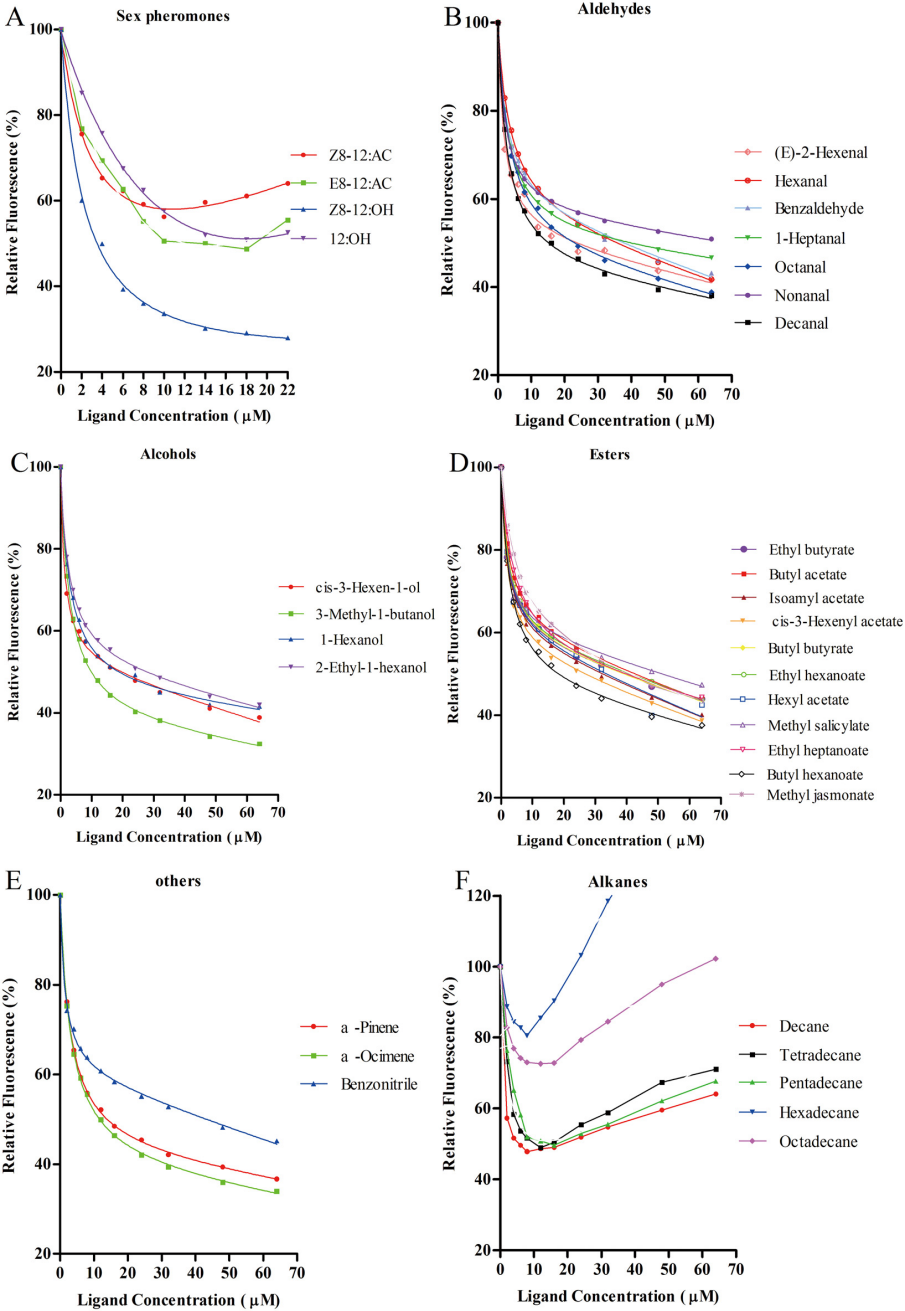
Fluorescence competitive binding curves of rGmolGOBP1 to sex pheromone components and host plant volatiles. A. rGmolGOBP1 to sex pheromone components; B, C, D, F. rGmolGOBP1 to aldehydes, alcohols, esters and alkanes; E. rGmolGOBP1 to terpenoids and benzonitrile. Mixtures of proteins and 1-NPN, at a 2 μM concentration, were titrated with 1 mM of each sex pheromone component and host-plant volatile to final concentrations of 0 μM to 22 μM and 0 μM to 64 μM, respectively. Relative fluorescence intensities are shown as a percent of the initial fluorescence without competitors.

**Fig 5:**
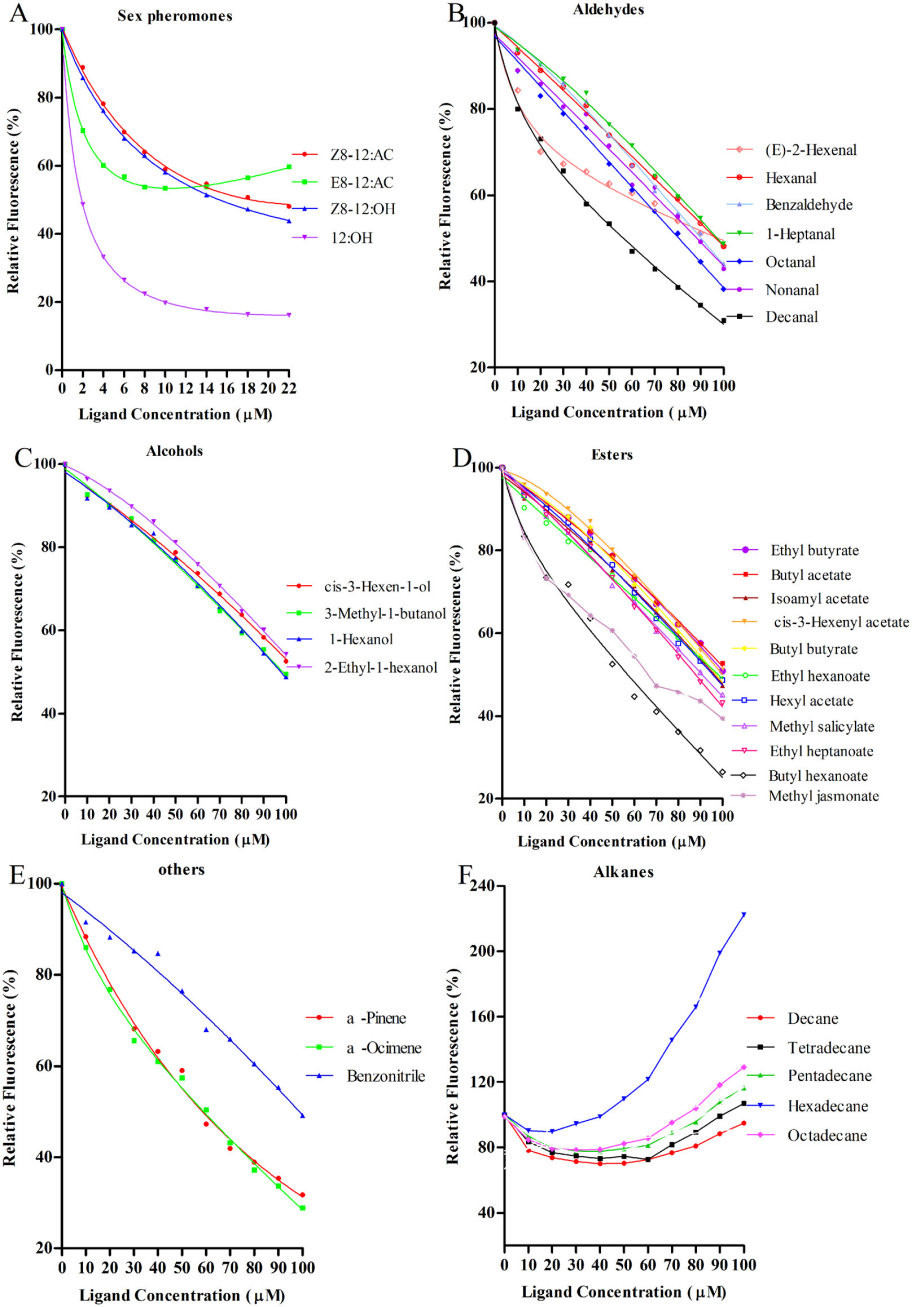
Fluorescence competitive binding curves of rGmolGOBP2 to sex pheromone components and host plant volatiles. A. rGmolGOBP2 to sex pheromone components; B, C, D, F. rGmolGOBP2 to aldehydes, alcohols, esters and alkanes; E. rGmolGOBP2 to terpenoids and benzonitrile. Mixtures of proteins and 1-NPN, at a 2 μM concentration, were titrated with 1 mM of each sex pheromone component and host-plant volatile to final concentrations of 0 μM to 22 μM and 0 μM to 100 μM, respectively. Relative fluorescence intensities are shown as a percent of the initial fluorescence without competitors.

Using 1-NPN as the fluorescent reporter, the binding affinities of two rGmolGOBPs with four sex pheromone components, (Z)-8-dodecenyl acetate, (E)-8-dodecenyl acetate, (Z)-8-dodecenyl alcohol and dodecanol, were evaluated in competitive binding assays (Table 2). rGmolGOBP1 exhibited strong binding affinities for the two pheromone components, (Z)-8-dodecenyl alcohol and (E)-8-dodecenyl acetate, with K_i_ values of 3.14 and 8.85 μM, respectively. However, rGmolGOBP1 did not bind to (Z)-8-dodecenyl acetate and dodecanol (Fig 4A). rGmolGOBP2 had the highest binding ability to dodecanol with K_i_ value of 1.22 μM, and also had medium binding affinities to (Z)-8-dodecenyl acetate and (Z)-8-dodecenyl alcohol with K_i_ values of 14.40 and 12.79 μM, respectively (Fig 5A).

**Table 2:** Binding affinities of ligands to GmolGOBP1 and 2 evaluated via competitive binding assays by using the fluorescent probe, 1-NPN.

Chemical compounds	GmolGOBP1		GmolGOBP2	
	IC_50_	K_*i*_	IC_50_	K_*i*_
Z8-12:AC	-	-	17.44±0.87	14.40
E8-12:AC	10.45±0.87	8.85	-	-
Z8-12:OH	3.85±0.41	3.14	15.49±0.52	12.79
12:OH	-	-	1.78±0.23	1.22
Cis-3-Hexen-1-ol	18.26±1.55	16.79	-	-
3-Methyl-1-butanol	11.61±0.81	10.68	-	-
1-Hexanol	21.37±1.80	19.65	-	-
2-Ethyl-1-hexanol	26.98±1.81	24.81	-	-
(E)-2-Hexenal	23.05±1.85	21.20	-	-
Hexanal	33.61±2.00	30.91	-	-
Benzaldehyde	34.97±3.28	32.16	91.35±2.87	75.41
Heptanal	37.15±4.50	34.16	-	-
Octanal	23.34±1.43	21.46	80.40±3.79	66.37
Nonanal	59.18±8.70	54.42	91.23±5.49	75.31
Decanal	17.35±1.30	15.96	51.33±3.18	42.37
Ethyl butyrate	36.07±2.88	33.17	-	-
Butyl acetate	39.91±2.66	36.70	-	-
Isoamyl acetate	28.95±2.18	26.62	-	-
Cis-3-Hexenyl acetate	24.31±1.71	22.36	-	-
Butyl butyrate	36.02±2.50	33.12	-	-
Ethyl hexanoate	38.10±3.36	35.04	-	-
Hexyl acetate	29.90±4.11	27.50	98.83±3.63	81.58
Methyl salicylate	49.77±6.48	45.77	93.66±5.55	77.31
Ethyl heptanoate	37.37±2.35	34.37	88.66±3.01	73.19
Butyl hexanoate	19.24±1.27	17.69	52.19±2.49	43.08
Methyl jasmonate	38.95±1.56	35.82	73.67±5.00	60.81
α-Pinene	16.15±1.22	14.85	51.65±7.11	42.64
α-Ocimene	13.53±0.79	12.44	53.38±4.91	44.06
Benzonitrile	40.39±1.95	37.14	-	-
Decane	5.64±0.69	5.19	-	-
Tetradecane	9.21±1.31	8.47	-	-
Pentadecane	12.41±1.87	11.41	-	-
Hexadecane	-	-	-	-
Octadecane	-	-	-	-

IC_50_. ligand concentration displacing half of the initial fluorescence intensity of the rGmolGOBPs/1-NPN complex. The inhibition constants (K_i_) were calculated from the IC_50_ values.

“-” means that the IC_50_ and K_i_ values were not calculated.

rGmolGOBP1 showed broad binding properties towards 30 host plant-derived volatiles emitted from peach and pear. Twenty-eight of thirty putative ligands succeeded in displacing 1-NPN from the GmolGOBP1/1-NPN complex by half at concentrations up to 60 μM. rGmolGOBP1 had strong binding affinities to small weight molecular aliphatic alkanes, particularly to decane and tetradecane with the k_i_ vaule of 5.19 and 8.47 μM, respectively (Fig 4F, Table 2). Length of the carbon chain of aliphatic alkanes had significant effect on binding affinity of rGmolGOBP1, decane and tetradecane exhibited outstanding binding affinities to rGmolGOBP1, pentadecane showed medium binding ability (with k_i_ vaule of 11.41 μM), while hexadecane and octadecane had very weak binding affinities. As to the phenomenon that binding affinities decreased with longer carbon chain of aliphatic alkane, it was the reason that the long carbon chains could not enter the binding pocket of GmolGOBP1. Except for aliphatic alkanes, cis-3-hexen-1-ol, 3-methyl-1-butanol, decanal, butyl hexanoate, α-pinene and α-ocimene also had medium binding activities to rGmolGOBP1 with K_i_ values of 16.79, 10.68, 15.96, 17.69, 14.85, and 12.44 μM, respectively (Fig 4B–4E, Table 2). rGmolGOBP2 exhibited very weak binding abilities to all tested ligands, and the K_i_ values were over 40 μM (Fig 5B–5F, Table 2).

### Effect of pH on binding affinity of rGmolGOBP2

**Fig 6:**
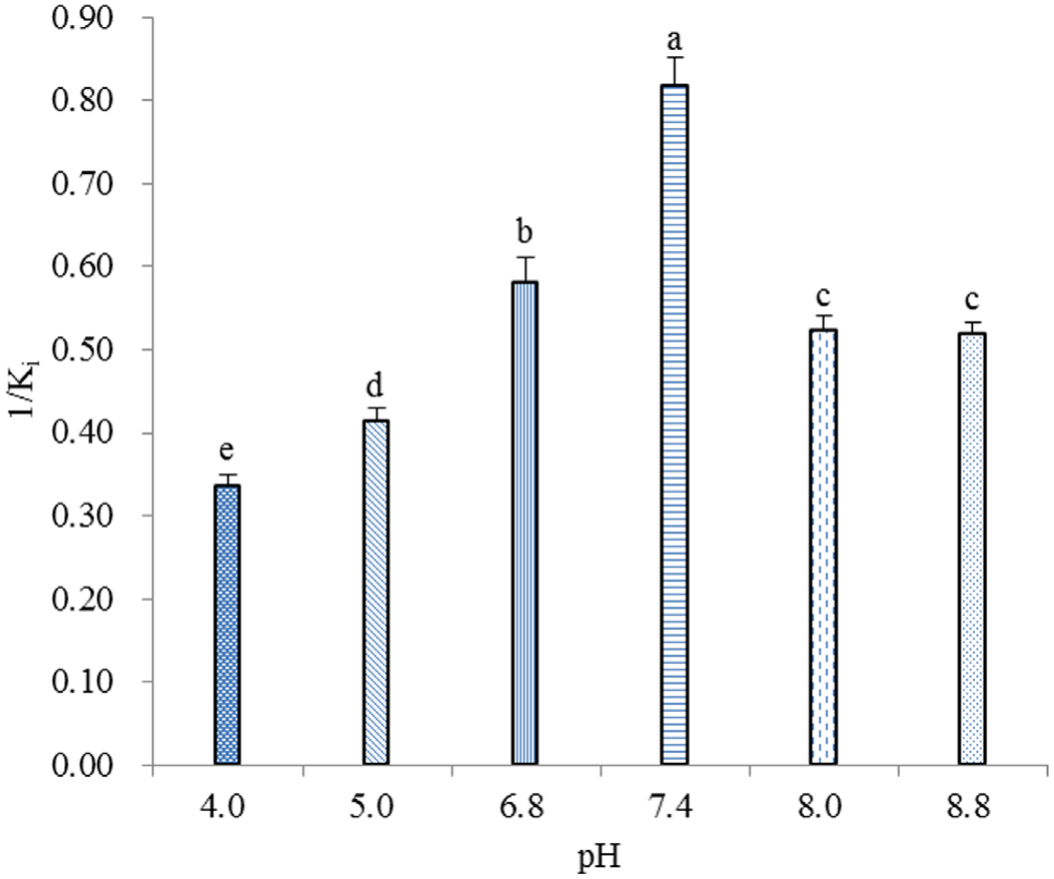
Effects of pH on the binding of rGmolGOBP2 with dodecanol. The rGmolGOBP2 and 1-NPN were both at 2 μM. The mixture solution was titrated with 1 mM of dodecanol to final concentrations of 14 μM. The K_i_ value was calculated as previously mentioned. The solutions were excited at 337 nm, and emission spectra were monitored between 370 and 550 nm. All data represent a mean of three independent measurements, and the bars denote mean±SEM. Values that not share the same letter have significant difference (S–N–K, P<0.05).

The effect of pH on the binding affinities of rGmolGOBP2 was investigated with the representative ligand dodecanol because of its specific binding to protein. Except between pH 8.0 and 8.8, the binding affinities of rGmolGOBP2 were significantly different among other pH (4.0, 5.0, 6.8, 7.4 and 8.0 (or 8.8)) (P<0.05), and the maximum value appeared at pH 7.4 (Fig 6), implying that the mechanism of ligand binding and release of GmolGOBP2 is related to a pH-dependent conformational transition.

### 3D model of GmolGOBP2 and molecular docking

**Fig 7:**
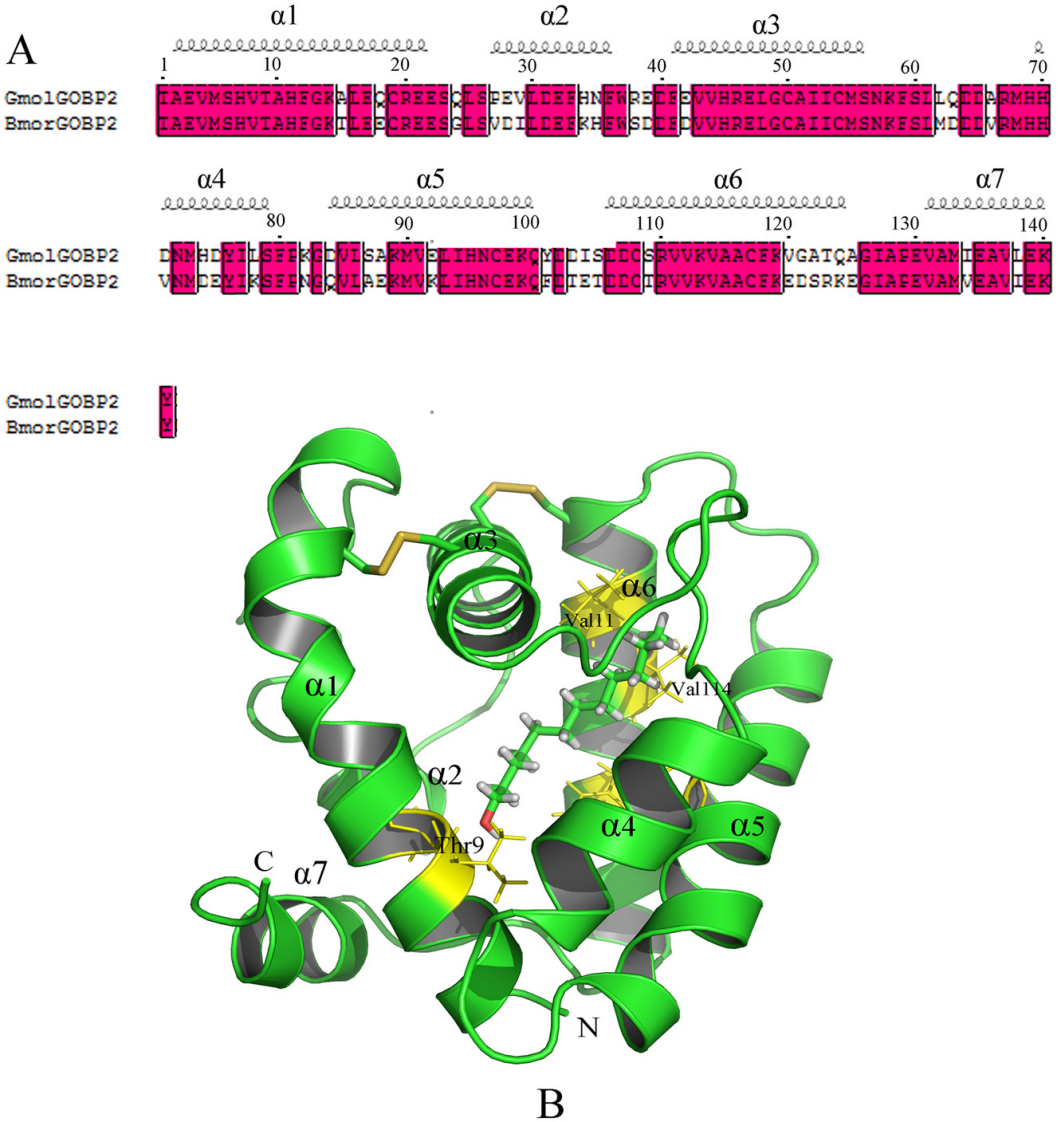
Modeled 3D structure and molecular docking experiments of GmolGOBP2. (A) Sequence alignment of GmolGOBP2 and BmorGOBP2. α-helices are displayed as squiggles. Strictly identical residues are framed and highlighted with a red background. (B) Overall structure of the GmolGOBP2 and the docking result. Three disulfide bridges are colored in yellow. N-terminal, C-terminal and α-helices are labeled. The top three potential key residues, Thr9, Val111 and Val114, are labeled in black font. Dodecanol is shown as a stick model with the hydroxyl oxygen in red.

By Meta serve searching, four structurally determined OBPs, including *Bombyx mori* OBP (BmorGOBP2), *Bombyx mori* PBP (BmorPBP), *Amyelois transitella* PBP (AtraPBP1) and *Antheraea polyphemus* PBP (ApolPBP) [17,32], were found to share sequence identity more than 30% compared to GmolGOBP2. The sequence similarity and coverage of GmolGOBP2 to BmorGOBP2 are highest, up to 74.47% and 100.00%, respectively (Fig 7A). We selected BmorGOBP2 as the structural template and built a 3D structural model of GmolGOBP2 using MODELER. After structural optimization, the verified score of the final GmolGOBP2 model by Profile-3D was 39.52, implying that the quality of the predicted GmolGOBP2 model was highly reliable.

The predicted 3D structure of GmolGOBP2 consisted of seven α-helices, six of which are located between residues 1–22 (α1), 27–36 (α2), 41–56 (α3), 70–79 (α4), 84–100 (α5) and 106–125 (α6). Three pairs of disulfide bridges connected Cys19 in α1 and Cys54 in α3, Cys50 in α3 and Cys108 in α6, and Cys97 in α5 and Cys117 in α6 (Fig 7B). C-terminal decapeptide segment (131–140) formed the seventh regular helix α7, which packed across the top of the N-terminal helix. In this model, a binding pocket was formed by α1, α3, α4, α5 and α6. Approximately half of the residues forming the pocket were observed to be hydrophobic (valine, alanine, leucine, isoleucine, proline, phenylalanine, etc). Many hydrophilic residues (asparagine, tyrosine, serine, etc.) were also present in the binding site of the pocket and probably participated in specific recognition by means of hydrogen bonding with hydroxyl oxygen of dodecanol.

In order to investigate the key binding sites of GmolGOBP2 with ligands, dodecanol was selected to dock with the predicted GmolGOBP2 model since it had the strongest binding affinity with protein. A lower value of interaction energy shows a stronger binding between GmolGOBP2 and dodecanol. All the top 20 residues with lower interaction energy to dodecanol were represented, including Met5, Val8, Thr9, Phe12, Phe33, Phe36, Phe37, Ile52, Leu62, Ala66, Arg67, Met68, Met90, Ile94, Glu98, Arg110, Val111, Val114, Ala115 and Phe118 (Table 3). Among these residues, some amino acids appeared to be potential key sites for the ligand-binding, such as threonine 9, which was possibly involved in the formation of a hydrogen bond between GmolGOBP2 and dodecanol. Located in the bottom of the dodecanol binding site, maybe valine 111 and 114 were related to the formation of a hydrogen bond between the carbonyl oxygen of other residues and the hydroxyl oxygen of dodecanol.

**Table 3:** The interaction energies between dodecanol and potential binding sites in the binding pocket of GmolGOBP2.

Residues	K_int_ (kcal/mol)	E_vdw_(kcal/mol)	E_ele_(kcal/mol)
A_MET5	-0.660	-0.841	0.181
A_VAL8	-2.997	-1.870	-1.127
A_THR9	-5.242	-1.936	-3.306
A_PHE12	-1.906	-1.634	-0.272
A_PHE33	-1.577	-1.656	0.079
A_PHE36	-2.275	-2.197	-0.078
A_PHE37	-1.592	-1.173	-0.419
A_ILE52	-1.837	-2.042	0.205
A_LEU62	-1.568	-1.588	0.020
A_ALA66	-0.690	-0.583	-0.107
A_ARG67	-1.290	-1.444	0.154
A_MET68	-1.359	-1.371	0.012
A_MET90	-1.116	-0.993	-0.123
A_ILE94	-2.516	-2.506	-0.010
A_GLU98	-1.857	-1.743	-0.114
A_ARG110	-0.433	-0.554	0.121
A_VAL111	-2.840	-2.665	-0.175
A_VAL114	-3.082	-3.088	0.006
A_ALA115	-2.229	-2.282	0.053
A_PHE118	-2.457	-2.672	0.215

K_int_, interaction energy; E_vdw_, Van der Waals energy; K_ele_, electrostatic interaction energy.

### Site-directed mutagenesis of GmolGOBP2 and binding specificities of mutants

**Fig 8:**
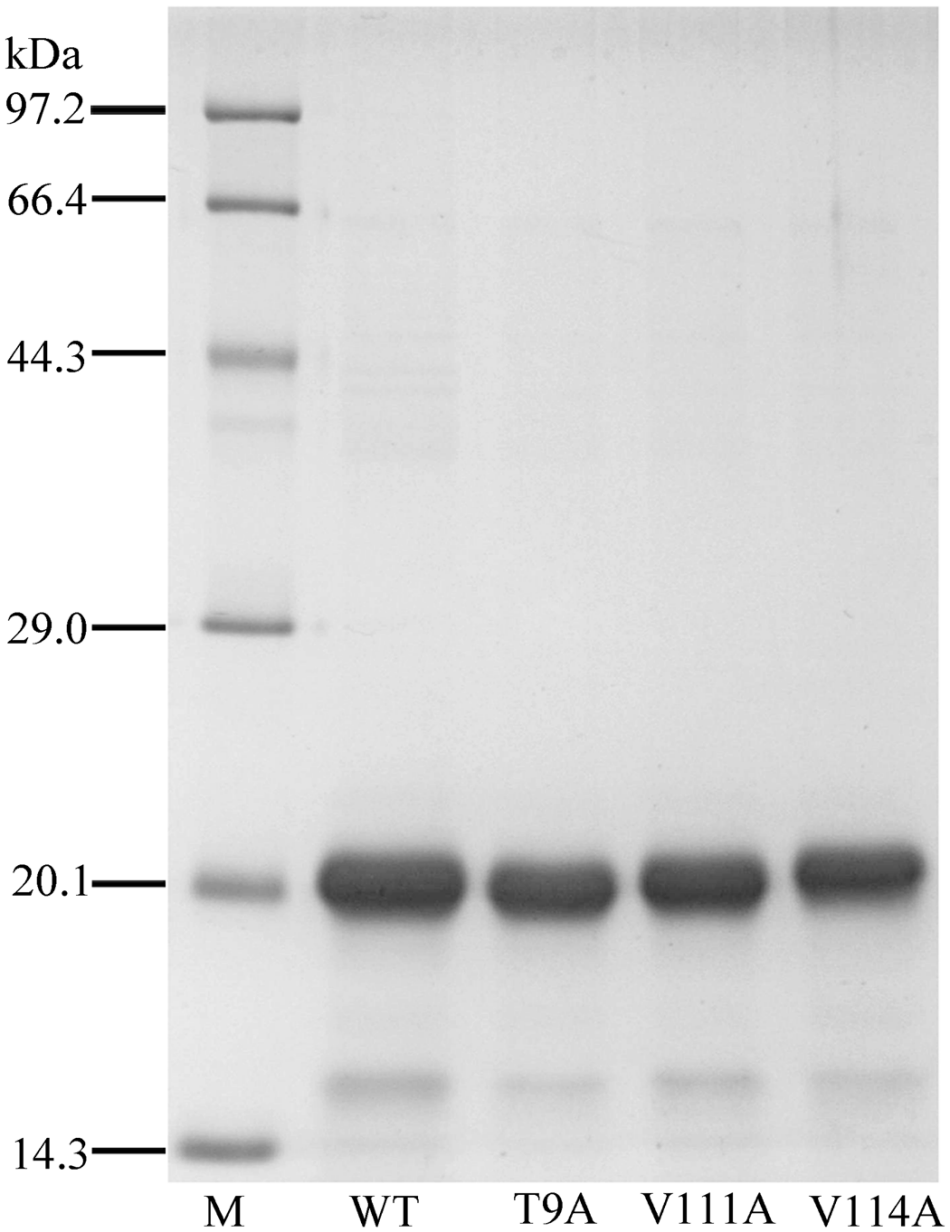
SDS-PAGE analysis of rGmolGOBP2 wild-type (WT) and mutants T9A, V111A and V114A.

**Fig 9:**
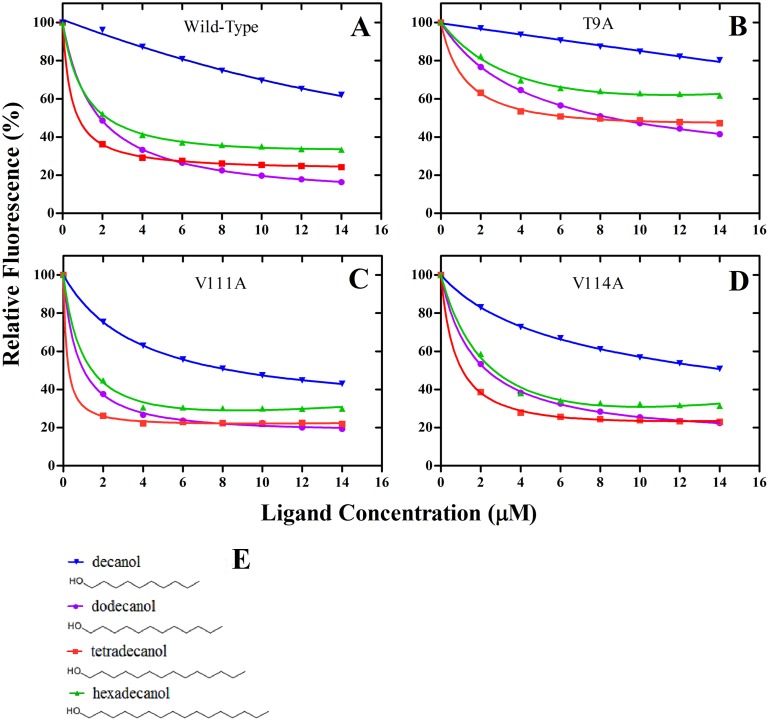
Comparison of the binding properties of GmolGOBP2 wild-type (A) and mutants T9A (B), V111A (C), V114A (D). The recombinant protein and 1-NPN were diluted to a fixed concentration of 2 μM. The mixture solution was titrated with 1 mM of each ligand to final concentrations of 0 μM to 14 μM. Relative fluorescence intensities are shown as a percent of the initial fluorescence without competitors. All data are an average of three independent measurements. Structures of ligands decanol, dodecanol, tetradecanol and hexadecanol (E) were plotted by ChemSketch.

Using site-directed mutagenesis, three potential key amino acid residues (Thr9, Val111 and Val114) were substituted with alanine, yielding the three mutants T9A, V111A and V114A. The analysis of SDS-PAGE showed that the mutants did not affect protein solubility; three mutant proteins were all present in the form of inclusion bodies, the same as wild-type rGmolGOBP2 (Fig 8). We investigated the affinities of the sex pheromone component dodecanol and analogs decanol, tetradecanol and hexadecanol to displace 1-NPN. The wild-type rGmolGOBP2 had the strongest binding affinity to tetradecanol, followed by dodecanol. The binding capacity to aliphatic alcohols was significantly reduced when the length of the carbon chain was less than C-10 or more than C-16 (Tables 2 and 4). T9A mutant showed a drastical decline in affinity to tetradecanol and hexadecanol and also showed a 3-5-fold decrease to dodecanol and decanol (Fig 9). In contrast, V111A and V114A mutants showed slightly lower affinities to dodecanol, and no differences were found to decanol, tetradecanol and hexadecanol (Table 4). It was verified that Thr9, a hydrophilic amino acid at the entrance of the binding cavity of GmolGOBP2, was one of the key binding sites of dodecanol and its analogs.

**Table 4:** Binding affinities of dodecanol and its analogs to rGmolGOBP2 wild-type and mutants.

Ligands	K_i_(μM)			
	Wild-Type	T9A	V111A	V114A
decanol	15.28±1.00	39.62±2.34	11.27±0.17	15.61±0.43
dodecanol	1.22±0.08	6.04±0.17	1.35±0.22	1.78±0.13
tetradecanol	0.36±0.05	5.75±0.86	0.30±0.16	0.37±0.14
hexadecanol	1.35±0.45	22.35±3.03	1.36±0.13	1.99±0.74

K_i_, dissociation constant. The values are the means of three independent experiments.

## Discussion

Many reports suggested that insect PBPs were located in the sensilla trichodea with specific binding to sex pheromones [19,20], and GOBPs were located in sensilla basiconica or sensilla trichodea, which were thought to recognize general odorants and other environmental semiochemicals [24,31]. With an increasing number of moth GOBPs and PBPs available, a lot of evidence showed that PBPs can bind to general odorants in addition to sex pheromone components and analogs [36,45]. Similar to PBPs, GOBPs also exhibited preference to special sex pheromone components besides host plant-derivated odorants [46,47]. GmolGOBP1 and 2 were generally distributed in s. basiconica labeled by anti-GmolGOBP1 and 2, and also involved in the detection of sex pheromone components, GmolGOBP2 even exhibited specific binding ability to dodecanol. Zhang et al [21] considered that the receptivity to odorant molecules is determined by two aspects: (i) abundant odor-sensitive ORNs; and (ii) the high binding affinity of PBP or OBP to odorant molecules. We speculated that the ORNs of s. basiconica containing GmolGOBP1 and 2 were sensitive to sex pheromone components (Z)-8-dodecenyl alcohol and dodecanol, respectively.

A previous study in our laboratory demonstrated that the transcript levels of GmolGOBP1 and 2 are highly represented in both male and female antennae [39], revealing that GmolGOBP1 and 2 are likely to be involved in olfactory chemoreception. In order to understand the physiological function and binding specificity of GmolGOBP1 and 2, fluorescence binding assays were conducted with four sex pheromone components of *G*.*molesta* [48] and 30 host plant volatiles as binding ligands [4,49–53]. rGmolGOBP1 has broad binding activities to various ligands including aliphatic alcohols, aldehydes, esters, alkanes, as well as terpenoids and aromatic compound derivatives, whereas rGmolGOBP2 showed very weak binding affinities to all tested host plant volatiles. rGmolGOBP1 and 2 had exhibited strong affinities to at least one of the sex pheromone components. Therefore, it was inferred that the GmolGOBP1 had dual functions in the detection of both volatile odorants and sex pheromone components, while GmolGOBP2 was only involved in minor sex pheromone component perception and recognition. Our results were consistent with previous studies in Lepidoptera species showing that *Manduca sexta* MsexGOBP2 had specific binding to sex pheromone component (6E,11Z)-hexadecadienyl diazoacetate [54], that *Chilo suppressalis* CsupGOBP2 showed positive binding only with sex pheromone (Z)-11-hexadecenal [46] and that LstiGOBP2 had a high binding affinity for E-11-tetradecen-1-yl acetate, a sex pheromone component of *Loxostege sticticalis* [55]. The *M*. *sexta* MsexGOBP2 and MsexPBP1 derived from gene duplications since these two genes were tandemly located on the chromosome and some arbitrary translocation event occurred [56]. Multi-sequences alignment of GmolOBPs showed that GmolGOBP2 shared higher sequence similarities with PBPs (the similarities to PBP1, PBP2, and PBP3 was 27.4%, 27.8% and 28.5%, respectively) than other OBP sequences [35–38] (S2 File). Combined with the GmolGOBP2 acted as the functions of PBPs, we speculated the *G*.*molesta* GOBP2 and PBPs emerged from gene duplications. We also found that there were some differences between GmolOBPs and GmolPBPs. For instance, GmolPBPs were primarily responsible for recognizing and transporting the major sex pheromone components (the K_i_ values of rGmolPBP1 for (Z)-8-dodecenyl acetate and (E)-8-dodecenyl acetate were 1.10 and 1.09 μM, respectively) [36], while the GmolGOBPs and GmolABPXs proteins acted in perception and recognition of minor sex pheromone components (the K_i_ values of rGmolGOBP1, rGmolOBP8 and rGmolOBP11 for (Z)-8-dodecenyl alcohol were 3.14, 5.59 and 3.05 μM, rGmolGOBP2 and rGmolOBP15 for dodecanol were 1.22 and 3.24 μM, respectively) [38], revealing the ligand-binding functions of each GmolOBP protein highly differentiated themselves during the long evolutionary process of this species.

Fluorescence competition assays further verified that dodecanol analogs also bind to rGmolGOBP2, and the C12-14 aliphatic alcohols were the optimal ligands. Dodecanol has been reported to be an attractant synergist that increased the frequency of male landing and a stimulant that induced mating behavior when the male and female were close to each other [48,57]. Combining this information with previous behavior tests and our fluorescence replacement experiments, we considered that protein GmolGOBP2 and dodecanol odors as well as analogs may play important roles in finding mates for adult *G*.*molesta*.

The pH effect on ligand binding and release revealed that GmolGOBP2 undergoes a pH-dependent conformational transition mechanism. rGmolGOBP2 showed a pronounced binding affinity with dodecanol at pH 7.4, while it displayed a weak ability at pH 4.0. Based on 3D structural modeling, a possible explanation is that C-terminal decapeptide segment forming helix α7 occupies the binding site in the hydrophobic binding pocket and inhibits the binding of pheromone molecules at low pH. The binding and release of GmolGOBP2-dodecanol complex perhaps was similar to those observed in BmorGOBP2, ApolPBP and AtraPBP1 [17,58,59]. Compared to the wild type rGmolGOBP2 protein, the T9A mutation showed lower affinities to dodecanol and its analogs. The most credible interpretation is that aliphatic alcohols cannot be recognized by the mutant and enters the binding cavity due to the loss of hydrogen bonding. V111A and V114A mutants showed a slightly decreased capacity to bind alcohols; this might be attributed to the insufficiency of the mutant to establish specific van der Waals interactions [8].

Since Sandler et al [32] first reported the 3D molecular model of *Bombyx mori* BmPBP, the conformational transitions from ligand-binding and -releasing have been analyzed for OBPs of several species [60,61]. In combination with the results in other reports, we considered that the hydrophilic resides at the entrance of the OBP binding cavity were conducive to initial ligand detection and recognition. For example, Ser52 and Thr57 of LUSH, the two hydrophilic amino acid residues located at the entrance of the binding pocket, were key binding sites for the formation of hydrogen bonds with hydroxyl of alcohol [33]. Moreover, Asn53 of ApolPBP1 and Ser56 of BmorPBP, the two hydrophilic amino acids at the opening of the binding cavities, were critical binding sites for specific binding to (6*E*,11*Z*)-hexadeca-6,11-dienyl-1-acetate and bombykol, respectively [32,58]. We speculated that the Thr9 of GmolGOBP2, located at the entrance of the binding pocket, is one of the crucial binding sites involved in the initial recognition of dodecanol.

## Supporting Information

S1 File The sources of four sex pheromones and 30 plant volatile standard chemical compounds used in the binding assays. (DOCX)

S2 File Sequence alignment of OBPs from *G*. *molesta*. (DOCX)
