# Supplementary material for: Binding Properties of General Odorant Binding Proteins from the Oriental Fruit Moth, Grapholita molesta (Busck) (Lepidoptera: Tortricidae)
Source: PLoS One. 2016 May 6;11(5):e0155096. doi: 10.1371/journal.pone.0155096 (PMC4859520; doi:10.1371/journal.pone.0155096)
Supplement: S1 File — (DOCX) [file pone.0155096.s001.docx]

| Chemical compounds | Molecular weight | Formula | Purity | Source |
| --- | --- | --- | --- | --- |
| **Sex pheromones** |  |  |  |  |
| (Z)-8-dodecenyl acetate | 226.36 | C14H26O2 | >95.0%(AR) | Bedoukian Research |
| (E)-8-dodecenyl acetate | 226.36 | C14H26O2 | >95.0%(AR) | Bedoukian Research |
| (Z)-8-dodecenyl alcohol | 200.34 | C12H24O | >98.0%(AR) | Bedoukian Research |
| dodecanol | 186.34 | C12H26O | >99.0%(GC) | Bedoukian Research |
| **Alcohols** |  |  |  |  |
| 3-Methyl-1-butanol | 88.15 | C5H12O | >98.0%(AR) | TCI |
| Cis-3-Hexen-1-ol | 100.16 | C6H12O | 98.0%(AR) | Sigma |
| 1-Hexanol | 102.18 | C6H14O | >98.0%(AR) | TCI |
| 2-Ethyl-1-hexanol | 130.23 | C8H18O | >99.0%(GC) | aladdin |
| **Aldehydes** |  |  |  |  |
| (E)-2-Hexenal | 98.15 | C6H10O | 98.0%(AR) | Alfa |
| Hexanal | 100.16 | C6H12O | >95.0%(AR) | TCI |
| Benzaldehyde | 106.12 | C7H6O | ≥99.5%(GC) | aladdin |
| Heptanal | 114.18 | C7H14O | 97.0%(AR) | aladdin |
| Octanal | 128.21 | C8H16O | 99.0%(AR) | aladdin |
| Nonanal | 142.24 | C9H18O | 95.0%(AR) | Sigma |
| Decanal | 156.26 | C10H20O | 97.0%(AR) | aladdin |
| **Esters** |  |  |  |  |
| Ethyl butyrate | 116.16 | C6H12O2 | 99.0%(AR) | aladdin |
| Butyl acetate | 116.16 | C6H12O2 | 99.0%(AR) | aladdin |
| Isoamyl acetate | 130.19 | C7H14O2 | ≥99.5%(GC) | aladdin |
| Cis-3-Hexenyl acetate | 142.20 | C8H14O2 | >97.0%(GC) | TCI |
| Butyl butyrate | 144.22 | C8H16O2 | >99.0%(GC) | aladdin |
| Ethyl hexanoate | 144.21 | C8H16O2 | 99.0%(AR) | aladdin |
| Hexyl acetate | 144.21 | C8H16O2 | 99.0%(AR) | aladdin |
| Methyl salicylate | 152.15 | C8H8O3 | ≥99.0%(GC) | Sigma |
| Ethyl heptanoate | 158.24 | C9H18O2 | ≥99.5%(GC) | aladdin |
| Butyl hexanoate | 172.27 | C10H20O2 | ≥99.5%(GC) | aladdin |
| Methyl jasmonate | 224.30 | C13H20O3 | 98.0%(AR) | aladdin |
| **Terpenoids** |  |  |  |  |
| α-Pinene | 136.23 | C10H16 | 98.0%(AR) | Sigma |
| α-Ocimene | 136.23 | C10H16 | ≥90.0%(AR) | aladdin |
| **Benzonitriles** |  |  |  |  |
| Benzonitrile | 103.12 | C7H5N | >99.0%(AR) | TCI |
| **Alkanes** |  |  |  |  |
| Decane | 142.29 | C10H22 | 98.0%(AR) | aladdin |
| Tetradecane | 198.39 | C14H30 | 98.0%(AR) | aladdin |
| Pentadecane | 212.42 | C15H32 | 98.0%(AR) | aladdin |
| Hexadecane | 226.44 | C16H34 | 98.0%(AR) | aladdin |
| Octadecane | 254.50 | C18H38 | 98.0%(AR) | aladdin |

S1 File. The sources of four sex pheromones and 30 plant volatile standard chemical compounds used in the binding assays
