# Supplementary material for: Binding Properties of General Odorant Binding Proteins from the Oriental Fruit Moth, Grapholita molesta (Busck) (Lepidoptera: Tortricidae)
Source: PLoS One. 2016 May 6;11(5):e0155096. doi: 10.1371/journal.pone.0155096 (PMC4859520; doi:10.1371/journal.pone.0155096)
Supplement: S2 File — (DOCX) [file pone.0155096.s002.docx]

S2 File. Sequence alignment of OBPs from *G. molesta*

|  | GOBP1 | GOBP2 | PBP1 | PBP2 | PBP3 | OBP 4 | OBP 6 | OBP 8 | OBP 10 | OBP 11 | OBP 12 | OBP 13 | OBP 15 | OBP 20 |
| --- | --- | --- | --- | --- | --- | --- | --- | --- | --- | --- | --- | --- | --- | --- |
| GOBP1 | 100 | 39 | 26 | 27 | 25 | 12 | 14 | 9.5 | 9.0 | 9.0 | 8.4 | 9.5 | 10.0 | 9.0 |
| GOBP2 |  | 100 | 27.4 | 27.4 | 25.8 | 10.5 | 13.2 | 10.0 | 10.5 | 7.9 | 6.8 | 10.0 | 9.5 | 8.4 |
| PBP1 |  |  | 100 | 34.2 | 34.7 | 11.1 | 11.6 | 9.0 | 9.0 | 11.1 | 12.1 | 10.0 | 12.1 | 6.3 |
| PBP2 |  |  |  | 100 | 39 | 10.5 | 9.5 | 7.4 | 8.4 | 6.8 | 9.5 | 9.5 | 7.4 | 8.4 |
| PBP3 |  |  |  |  | 100 | 8.4 | 12.1 | 7.9 | 12.1 | 6.8 | 7.9 | 7.9 | 8.4 | 5.8 |
| OBP 4 |  |  |  |  |  | 100 | 12.1 | 15.3 | 29.5 | 14.7 | 40.0 | 14.7 | 8.4 | 11.6 |
| OBP 6 |  |  |  |  |  |  | 100 | 13.7 | 10.5 | 10.0 | 7.9 | 9.5 | 10.5 | 9.5 |
| OBP 8 |  |  |  |  |  |  |  | 100 | 15.8 | 44.2 | 15.8 | 15.8 | 11.1 | 14.2 |
| OBP 10 |  |  |  |  |  |  |  |  | 100 | 14.2 | 31.1 | 15.8 | 7.9 | 10.5 |
| OBP 11 |  |  |  |  |  |  |  |  |  | 100 | 14.2 | 18.4 | 9.5 | 15.3 |
| OBP 12 |  |  |  |  |  |  |  |  |  |  | 100 | 17.4 | 9.5 | 13.7 |
| OBP 13 |  |  |  |  |  |  |  |  |  |  |  | 100 | 11.5 | 15.8 |
| OBP 15 |  |  |  |  |  |  |  |  |  |  |  |  | 100 | 7.9 |
| OBP 20 |  |  |  |  |  |  |  |  |  |  |  |  |  | 100 |
